# Molecular Characterization of the Coat Protein Gene of Greek Apple Stem Pitting Virus Isolates: Evolution through Deletions, Insertions, and Recombination Events

**DOI:** 10.3390/plants10050917

**Published:** 2021-05-03

**Authors:** Matthaios M. Mathioudakis, Varvara I. Maliogka, Thierry Candresse, Osmar Nickel, Thor Vinicius Martins Fajardo, Daria Budzyńska, Beata Hasiów-Jaroszewska, Nikolaos I. Katis

**Affiliations:** 1 Plant Pathology Laboratory, Institute of Olive Tree, Subtropical Plants & Viticulture, ELGO-DIMITRA, Karamanlis Ave. 167, Gr-73134 Chania, Greece; 2 Laboratory of Plant Pathology, School of Agriculture, Faculty of Agriculture, Forestry and Natural Environment, Aristotle University of Thessaloniki, Gr-54124 Thessaloniki, Greece; vmaliogk@agro.auth.gr (V.I.M.); katis@agro.auth.gr (N.I.K.); 3 INRAE, UMR BFP, University of Bordeaux, CS20032, CEDEX, 33882 Villenave d’Ornon, France; thierry.candresse@inrae.fr; 4 Embrapa Uva e Vinho, 95.701-008, Bento Gonçalves, Rio Grande do Sul 96200, Brazil; osmar.nickel@embrapa.br (O.N.); thor.fajardo@embrapa.br (T.V.M.F.); 5 Department of Virology and Bacteriology, Institute of Plant Protection-National Research Institute, ul. Wł. Węgorka 20, 60-318 Poznań, Poland; d.budzynska@iorpib.poznan.pl (D.B.); b.hasiow@iorpib.poznan.pl (B.H.-J.)

**Keywords:** ASPV, CP-specific RT–PCR, pome fruits, botanical pears, variants, CP variability, indels, recombination

## Abstract

A RT–PCR assay developed to amplify the full coat protein (CP) gene of apple stem pitting virus (ASPV) was evaluated using 180 Greek apple and pear samples and showed a broad detection range. This method was used to investigate the presence of ASPV in quince in Greece and showed a high incidence of 52%. The sequences of 14 isolates from various hosts with a distinct RFLP profile were determined. ASPV population genetics and the factors driving ASPV evolution were analyzed using the Greek ASPV sequences, novel sequences from Brazilian apple trees and Chinese botanical *Pyrus* species, and homologous sequences retrieved from GenBank. Fourteen variant types of Greek, Brazilian and botanical isolates, which differ in CP gene length and presence of indels, were identified. In addition, these analyses showed high intra- and inter-group variation among isolates from different countries and hosts, indicating the significant variability present in ASPV. Recombination events were detected in four isolates originating from Greek pear and quince and two from Brazilian apples. In a phylogenetic analysis, there was a tendency for isolates to cluster together based on CP gene length, the isolation host, and the detection method applied. Although there was no strict clustering based on geographical origin, most isolates from a given country tended to regroup in specific clusters. Interestingly, it was found that the phylogeny was correlated to the type, position, and pattern of indels, which represent hallmarks of specific lineages and indicate their possible role in virus diversification, rather than the CP size itself. Evidence of recombination between isolates from botanical and cultivated species and the clustering of isolates from botanical species and isolates from cultivated species suggest the existence of a possible undetermined transmission mechanism allowing the exchange of ASPV isolates between the cultivated and wild/ornamental hosts.

## 1. Introduction

Apple stem pitting virus (ASPV) is one of the most widely distributed viruses of pome fruits worldwide [1]. It is a positive single-stranded RNA (ssRNA) virus with a genome of approximately 9.200–9300 nucleotides harboring five open reading frames (ORFs) and is classified in the genus *Foveavirus* in the Betaflexiviridae family [2,3]. The virus dissemination is likely due to noncertified propagative material and grafting, as no vector is currently known [4]. In Greece, pome fruits are very important and are mainly cultivated in the central and northern parts of the country. Although ASPV is frequently detected in a symptomless form, it is also associated with economically important diseases, such as quince fruit deformation, epinasty and decline of Spy 227 and pear vein yellows [2,5,6,7,8]. Moreover, the green crinkle disease in apples and a severe quince disease have been attributed to a distinct variant of ASPV, known as apple green crinkle-associated virus (AGCaV) [9,10]. Recent reports have expanded the known natural host range of ASPV, with its detection in ornamental and wild Rosaceae species and an exceptional report in cherries [2,11,12,13,14,15,16,17], further suggesting the potential existence of (a) yet unidentified transmission mode(s). In Greece, ASPV was first identified by molecular assays in apple, pear and quince [5,6], and later on, noncultivated species (ornamental and wild quince and pear) were also reported as natural hosts of ASPV [14].

ASPV shows an extremely high infection rate (91%) in apple trees in Greece and a lower one in pears (51%), with nucleotide (nt) sequence divergence levels of up to 29% in the conserved RNA dependent RNA polymerase (RdRp) gene of different virus isolates [13]. A range of other studies has illustrated the high genetic diversity existing in the ASPV coat protein (CP) gene, including in viral populations from single hosts [18,19,20,21,22,23,24]. The study and knowledge of the genomic variability of ASPV have important diagnostic implications as there are reports of the failure of serological and molecular detection procedures for routine diagnostics for some isolates/samples [20,25,26]. Recombination also plays an important role in RNA virus evolution and may give rise to new genotypes [27], and indeed, the availability of ASPV sequences has led to detecting numerous recombination events in its CP gene [20,24,28,29]. In conclusion, due to its high genetic variability in addition to identifying new strains and/or recombinant isolates, ASPV remains a challenge and potential threat to the pome fruit and nursery industries.

In this study, a two-step RT–PCR assay was developed using degenerate primers for specific ASPV detection. This technique allows the amplification of the full-length CP gene, together with part of the 3΄ untranslated region (3′UTR). Evaluation of this method using 180 ASPV apple and pear isolates showed its wide detection range. Moreover, a survey conducted in quince orchards showed the high incidence of ASPV in this host in Greece. After an RFLP analysis, 14 diverse ASPV isolates from apple, pear and quince were selected, and their complete CP gene sequence was determined. New ASPV CP sequences reported here from cultivated and noncultivated hosts were analyzed to provide further insights into possible evolutionary relationships between ASPV isolates from different hosts and origins and describe the potential forces leading to the ASPV genetic population structure.

## 2. Results

### 2.1. Development of a Specific RT–PCR Assay for ASPV Detection

The specific RT–PCR assays developed in this study, using an oligo d(T)_18_ for cDNA synthesis and different primer pairs comprised a common degenerate sense primer and of different degenerate antisense primers, successfully detected a range of ASPV isolates. The size of the expected amplicons ranged from 1220 to 1370 bp, depending on the primer pair used, corresponding to the full CP gene and different partial parts of the 3΄UTR. The efficiency of the methods was evaluated using a total of 180 ASPV isolates from apple (123 isolates) and pear (57 isolates) previously identified by a highly sensitive and broad-spectrum nested RT–PCR amplifying part of the RdRp gene [13]. The ASPV CP-3΄UTR region was successfully amplified in a total of 121 samples (89 from apple and 32 from pear). More specifically, 96 ASPV isolates (~1260–1370 bp) were detected by the PCR using the SPCPdw1 antisense primer, whereas 99 isolates (~1220–1320 bp) were detected by the PCR using the SPCPdw2 primer. A total of 74 ASPV isolates detected using the SPCPdw2 primer were also detected using SPCPdw1. No amplicons were detected when the oligo d(T)_28_ was used instead of the ASPV-specific antisense primers. All the 121 ASPV isolates detected using one or the other antisense primers were also detected when using them simultaneously, suggesting this as a suitable ASPV CP-specific RT–PCR assay.

### 2.2. Detection and Incidence of ASPV in Quince in Greece

The above method, which simultaneously uses both antisense primers in a single PCR reaction, was used to evaluate the presence and incidence of ASPV in Greek quince samples. ASPV was detected in 52% (25/48) of the tested quince samples from eight of the 10 surveyed districts (Table S1). As in apple and pear samples, no amplicons were detected when using the oligo d(T)_28_ primer. ASPV was detected in 24 of the 25 samples showing typical symptoms of quince fruit deformation and in a single additional sample from an asymptomatic tree from the Larissa district.

### 2.3. Preliminary Analysis for the Presence of ASPV Variants by RFLP

**Figure 1:**
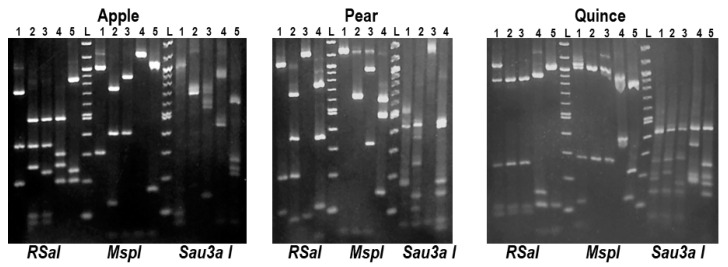
RFLP analysis of amplified PCR products using the *MspI*, *RsaI* and *Sau3AI* enzymes. Apple: lanes 1 to 5 represent, respectively isolates AGK69, AGK67, AUM109, AFL107 and AFT137. Pear: lanes 1 to 4 represent, respectively, isolates PCK74, PKF82, PHL193 and PDT95. Quince: lanes 1 to 5 represent QUL10, QAT4, QUK18, QUM155 and QGK58, respectively. L: DNA molecular marker 100 bp.

RFLP analysis was used to identify diverse ASPV variants using the *MspI*, *RsaI* and *Sau3AI* enzymes. The amplicons from a total of 146 ASPV isolates from apple (89), pear (32) and quince (25) were used in the RFLP analysis. The results showed the existence of different digestion patterns (data not shown). In apple samples 15 such patterns were identified, whereas 9 and 12 patterns were identified in pear and quince samples, respectively. Considering the RFLP profile of the isolates, the host and the district of origin, 14 ASPV isolates (five from apple, four from pear and five from quince) were selected for further analysis (Figure 1). In the case of quince, one of the five chosen samples was collected from the asymptomatic one. The names of the selected isolates are provided in Table 1.

### 2.4. Molecular Characterization of the Complete CP and Partial 3΄UTR Sequences of the 14 Selected Greek Isolates

Amplicons from the 14 selected isolates were cloned and the sequence of their CP gene and partial 3΄UTR determined by Sanger sequencing on both strands of the cloned cDNAs. The CP genes of these isolates showed a length heterogeneity of up to 108 nt, with their size varying from 1125 to 1233 nt (375 to 411 amino acids (aa)) (Table 1). The multiple alignments of the obtained sequences showed that the variability in CP length was due to 4 indels variations (Table 1) uncorrelated to the area of origin.

**Figure 2:**
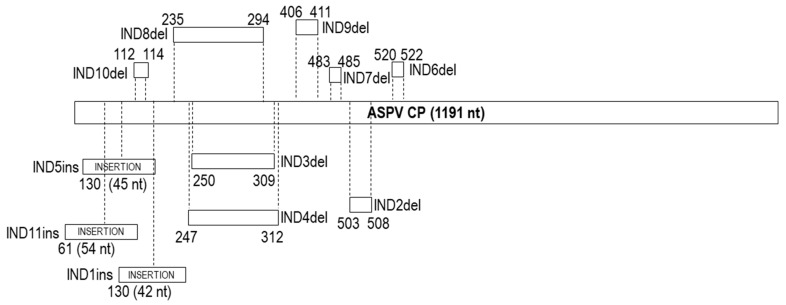
Schematic presentation for the localization of indels (designated as IND) identified in the ASPV CP gene of isolates used in this study. Indels type as deletions (marked as del) or insertions (marked as ins). Positions correspond compared to the 1191 nt CP size reference gene.

As compared to the most frequent ASPV CP length of 1191 nt (5 apples, 3 pears), in two isolates (PKF82, QUL10; 1233 nt), an insertion of 42 nt was found, designated as IND1ins (Figure 2, Figure S1). The quince isolates showed high length heterogeneity due to three different deletions of, respectively 6 nt (IND2del) (QUK18, QUM155; 1185 nt), 60 nt (IND3del) (QGK58; 1131 nt) and 66 nt (IND4del) (QAT4; 1125 nt) (Figure 2). The length of the partial 3΄UTR region ranged from 124 to 129 nt, while three isolates (PHL193, QUL10, QGK58) were successfully amplified only using primer SPCPdw1, and the partial 3΄UTRs were only 85 to 87 nt long.

The multiple alignments of 484 ASPV CP gene sequences (429 from the GenBank, 14 from Greek isolates, 24 from botanical *Pyrus* and 17 from Brazilian apples) showed that the Greek, botanical and Brazilian isolates shared a similar range of CP lengths (Table 1), with most ASPV isolates CP genes ranging from 1104 to 1245 nt (extremes 1092 and 1248 nt). Taking as reference 1191 nt most frequent length (184 out of 484 isolates), the analysis showed the presence of a high number of indel polymorphisms (30 additional different indels). The indels observed in Greek isolates were identified again in isolates of the same CP length: IND1ins was also observed in 38 isolates (35 pears, one apple, two botanical: *P. ussuriensis*, *P. pyrifolia*) (Figure S1), IND4del in 17 pear isolates (16 cultivated, one botanical: *P. pyrifolia*), IND3del in 11 pear isolates, and IND2del in 34 isolates (29 pears, two apples, one Brazilian apple, 2 botanical: *P. ussuriensis*, *P. pyrifolia*) (Table 1), suggesting that there is no or only limited correlation between the presence of particular indels and either the country of origin or the isolation host.

Many unique point mutations were also identified among all isolates (19 in Greek, 13 in botanical, 9 in Brazilian). Interestingly, in the AUM109 apple isolate, two point mutations (unique insertion of a C_444_ nucleotide and an nt_478_ deletion) cause creating a unique motif of 11 aa (ATWCQSLNS-AS) compared to all other isolates. The sequences used in this study were deposited in GenBank under accession numbers MW810244-MW810257 (Greek) and MW842988-MW843004 (Brazilian).

### 2.5. Molecular Variability Analysis of ASPV Isolates

A variability analysis among the Greek isolates showed a nucleotide divergence range of 3–29% and 6–26% for the corresponding CP aa sequences, with aa variation mainly distributed in the CP N-terminal half. It is noteworthy that some of these values fall outside of the 20% aa divergence used as one of the species molecular thresholds in the Betaflexiviridae family [30]. The highest identities were shared between same-host isolates: 97% nt identity between two quince isolates (QUK18/QUM155), 95% between two apple isolates (AGK69/AUM109) and 92% between two pear isolates (PHL193/PCK74). Among different hosts, the highest identities were between the QUL10 and PKF82 isolates (90% nt identity), whereas three apple isolates (AGK67, AFT137, AFL107) were the most distantly related with either the PKF82/QUL10 isolates or the QAT4/QGK58 isolates, showing 29% nt divergence.

The overall results of the complete CP sequences comparison between the 484 selected ASPV sequences (GenBank plus new sequences reported here) showed a very large divergence range of 6–37% nt identity and of 7–33% aa identity in the encoded CP. All the Greek isolates shared extreme nt divergence values of 34–37% with the following five isolates: FJ12 (pear, USA), Brae2 (apple, Brazil), N (apple, India), LYC (pear, China) and XJ (hawthorn, China) with the highest divergence of 37% observed between QAT4 and the Chinese XJ isolate from hawthorn. Apart from the extremely divergent isolates mentioned above, the Greek isolates also shared high nt divergence levels of 30–32% with isolates from cultivated species (pear: KY176819, JX673822, JX673821; apple: KY081215, KY081220) originating from Poland and China, with two new Brazilian isolates (Royal Gala ct5130 and ct1102), and with the isolates from botanical species *P. pyrifolia* ct207 (SRX5726450) and *P. ussuriensis* ct1418 (SRX670013). Considering all these data, it is generally clear that isolates from one host are not necessarily less divergent from different hosts.

### 2.6. Recombination Analysis

Changes in the phylogenetic relationships of a virus, when considering different genome regions, are often indicative of recombination events [9]. The existence of recombination events in the CP gene of ASPV has been reported previously [20,24,28]. We analyzed the potential recombination events in the CP gene using the dataset of 124 selected isolates described above, utilizing the RDP4 program. Only those events indicated by five or more programs incorporated in RDP4 were considered significant. The analysis revealed several potential recombination events listed in Table 2. In particular, potential recombination events were detected in three Greek isolates from quince and pear, whereas no recombination events were detected in the CP gene of apple isolates. These events were identified in QUL10 with KY176823 (apple) and KY243369 (pear) isolates as potential parents, and in PCK74 (same potential recombination event also present in pear isolate PHL193) with KY176815 (pear) and KY429181 (pear) as potential parents.

Potential recombination was also detected between the botanical *P. ussuriensis* ct54 isolate (SRX670013) with the JX673812 and JX673811 pear isolates as potential parents, and this event was also present in the closely related HM325767 pear and KY176808 apple isolates. Moreover, potential recombination events were detected in two Brazilian apple isolates. The Mishima ct15 isolate was identified as a potential recombinant of KR815875 (apple) and AF345892 (pear), and the same event was also present in KF319056 apple isolate. Interestingly, a potential recombination event was detected between Royal Gala ct1670 (recombinant) with the closely related AGCaV (HE963831) and the Brazilian Braeburn ct3334 apple isolates. The results of the RDP4 analysis were also confirmed by the SplitsTree4 decomposition phylogenetic networks (Figure S2).

### 2.7. Phylogenetic Relationships among ASPV Isolates: Potential Effect of CP Size, Host and Country of Origin, and of Virus Detection Method

The ASPV CP full-length nt sequences of the 14 Greek isolates were used to construct a neighbor-joining (NJ) tree in MEGA X (Figure S3), in which they were shown to regroup based on their host, with all apple isolates grouped together and quince and pear isolates forming a separate cluster.

A maximum-likelihood (ML) tree was constructed in MEGA X using the most likely substitution model GTR+*G*+*I* to infer the phylogenetic relationships among the 124 selected APSV CP nt sequences (Figure S4). In this analysis, which includes a much broader diversity of ASPV isolates, three main clusters (cluster I with its five subclusters (a–e); cluster II with three subclusters (f–h); cluster III with two subclusters) (Figure S5) are observed. This analysis shows a tendency for isolates to group according to their CP size, with some exceptions representing single isolates of unique CP sizes (Figure S6). The Greek isolates grouped in six of the subclusters from two of the three main clusters (apple: two subclusters (cluster II); pear: two subclusters (cluster I); quince: three subclusters (cluster I)) (Figures S4 and S5), confirming the significant ASPV diversity present in Greece. It is noteworthy that cluster III included all the highly divergent isolates from cultivated species (apple, pear) and close to two-thirds (14 out of 24) of the isolates from botanical pear species, which show six different CP sizes (1221 nt, 1218 nt, 1212 nt, 1200 nt, 1197 nt, 1194 nt) (Table 1).

**Figure 3:**
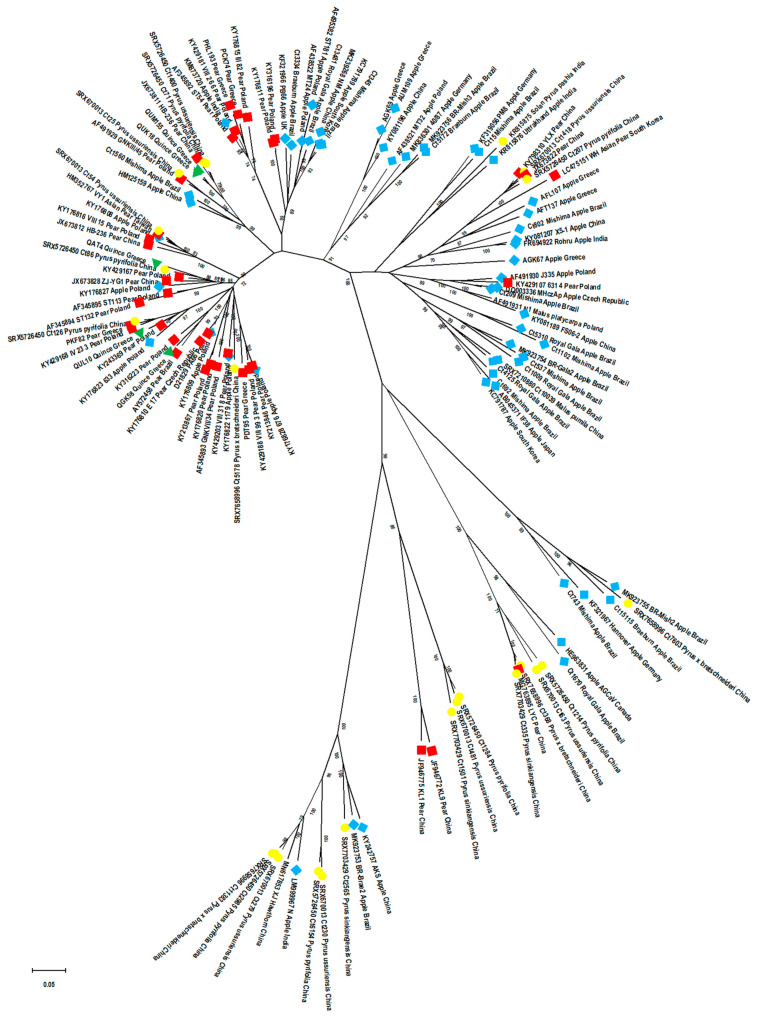
The ML phylogenetic tree (radical shape) of the CP nucleotide sequences, generated using MEGA X. The isolates are indicated by their accession number or isolate name, host and country of origin. The isolates are color-coded based on the host of origin; green: quince, light blue: apple, red: pear, yellow: botanical pears. Branches with <70% bootstraps were collapsed. The scale bars represent a genetic distance of 0.05. The classical shape tree can be examined in Figure S4.

To determine whether ASPV CP gene molecular variability is structured according to the host of origin, isolates in the ML tree were color-coded (apple: light blue, pear: red, quince: green, botanical *Pyrus*: yellow). The results showed a clear, although imperfect, segregation of isolates according to their isolation host (Figure 3), and this effect was even stronger at the subcluster level. In particular, cluster II is much enriched in apple isolates and contains very few isolates from other hosts, while cluster I gathers most of the pear isolates (30 out of 37) and all 5 quince isolates, while most apple isolates of cluster I (9 out of 17) are grouped in a specific subcluster (e). The majority (92%) of botanical *Pyrus* isolates are often separated from those from other hosts, with a tendency to group only in some subclusters (a, d, h, cluster III). These findings were confirmed by calculating the average intra-group and inter-group divergence values for host-based groups (Table 3). The isolates from botanical pear species are the most divergent isolates (inter-group) but also the most diverse group (intra-group divergence 0.298 ± 0.008) followed by apples (intra-group divergence 0.248 ± 0.008) with a statistically significant difference (Mann–Whitney–Wilcoxon test, *p* = 0).

A similar color-coding approach (China: green, Poland: pink, Brazil: red, Greece: blue, Rest of world: orange) and computation of intra-group/inter-group divergence was then used to evaluate whether the country of origin of the isolates similarly influenced viral diversity structuring. The results showed that contrary to the host of origin, no clear pattern emerged when considering the country of origin (Figure S7), despite the observation that cluster III does not contain any Polish or Greek isolates. Chinese isolates, representing the largest ensemble, and Brazilian isolates can be found in almost all subclusters with regrouping within subclusters based on the host of origin (Chinese: e, f, h, cluster III, Brazilian: a, b, h, cluster III). The calculation of the average pairwise genetic distances (Table 3) confirmed that Chinese isolates showed the highest intra-group value (0.290 ± 0.008) followed by the Brazilian isolates (0.256 ± 0.007), these values being significantly different in a Mann–Whitney–Wilcoxon test (*p* = 0). Polish and Greek isolates showed lower intra-group diversity, which is likely due to their presence in fewer subclusters and of their absence from cluster III. When considering between-country divergence (inter-group genetic distances), the Chinese and Brazilian isolates were the most divergent from other groups (Polish, Greek or rest of the world isolates).

As the new ASPV CP sequences reported here were determined either following RT–PCR amplification or unbiased HTS, we also investigated whether there is any correlation between phylogeny and how the sequences were obtained. The ML tree was thus color-coded with red for PCR-derived sequences (square for sequences obtained using one primer pair and circle for full-genome characterized isolates obtained by primer walking) and green for HTS-derived ones. The results show that cluster III does not contain any PCR-derived CP gene sequences, as these are only distributed between clusters I and II (Figure S8). These findings suggest that the absence of PCR-derived sequences in cluster III may result from an inability of detection primers to amplify these divergent variants or from another bias.

To investigate whether a similar clustering of ASPV isolates could be obtained when using the CP aa sequences instead of the nt sequences of the CP gene, an ML tree was constructed in MEGA X using the Jones–Taylor–Thornton (JTT)+*F*+*G*+*I* model to infer the phylogenetic relationships among the 124 selected APSV CP isolates (Figure S9). The analysis showed a stable existence of the same main clusters I-III, as shown in the nt-based tree, constituted by almost identical subclusters. Interestingly, the apple isolates in the subcluster e of the ML nt-tree were clearly grouped in a separate subcluster (d) in the ML aa-based tree. As was expected, these results fully confirm and support even further the findings obtained using the CP gene nt-based phylogenetic analysis.

### 2.8. Evolutionary Networks

For the study of evolutionary relationships between ASPV isolates, parsimony phylogenetic networks were created using the same alignment of 124 selected CP full-length nt sequences. The network obtained showed a star-radiation shape. This suggests the existence of a common origin of the isolates and their further diversification during evolution through mutations and recombination events. This hypothesis follows the phylogenetic relationships between isolates from different origins and CP size, as described above. The analysis revealed that all isolates grouped together with either QUL10 or *P. ussuriensis* ct54 or Mishima ct15 or Royal Gala ct1670 share a common origin and diversified by recombination events confirming the previous results (Figure S10). In contrast, the pear isolates PCK74 and PHL193, in which recombination events were also detected, occupied single tips indicating that the recombinants and parental isolates do not have a common origin.

### 2.9. Characterization of Insertions/Deletions (Indels): Type, Position, Variability and Their Impact on Population Structure

A correlation with a specific type of indel (deletion) and the phylogenetic clustering of isolates was shown in a previous study of Polish isolates, in which various ASPV CP-length isolates were used [24]. In the present study, the length of 1191 nt was used as a reference CP size for indels description, as it represents the largest number of isolates (including five apple and three pear Greek isolates, 11 Brazilian apple isolates and two botanical isolates studied here). To determine whether the tendency to phylogenetically regroup isolates according to their CP size is impacted by indels type (deletion/insertion), position and variability, the determined indels in the ML nt-based tree were color-coded (green: IND1ins, pink: IND2del, orange: IND3del, blue: IND4del, brick red: IND5ins, light blue: IND8del, purple: IND9del, gray: IND10del) (Figure S4). As expected, the results confirmed that the existence of CP size-based groups is another expression of a correlation with the type of indels and phylogenetic position of same-CP size isolates. This was the case for all same-CP size isolates sharing a given indel, such as, for example, IND4del, IND3del, and IND2del, which were grouped, respectively, into subclusters b, a, and d (Figure S4). Exceptionally, the isolates of 1233 nt CP size, although they possess the same IND1ins indel, are found in several subclusters (a, c), which could be due to the variability observed in the inserted sequence (Figures S1 and S4).

It is, however, interesting to note that same-CP size isolates that have indels of the same length but located at a different place within the CP gene are not tightly associated in the phylogenetic tree (e.g., 1185 and 1131 nt CP-length isolates, Figure S4), which indicates that is not CP size per se that is correlated with phylogeny and ASPV evolution, but rather specific indels, which mark specific lineages. This was observed in two cases. The isolates with a CP gene of 1131 nt differ by 60 nt deletions from the canonical 1191 nt CP gene due to possessing either IND3del or IND8del (Figure 2, Table 1). The identity of this 60 nt deletion drives the phylogenetic grouping of isolates sharing IND8del into a different subcluster (e) than those having IND3del (Figure S4). The same is observed for isolates with a CP gene of 1185 nt, which differ from the canonical reference gene by one of three 6 nt deletions (IND2del, IND9del or IND10del, Figure 2, Table 1). Again, such isolates are found to be regrouped in different subclusters depending on the particular indel they harbor (Figure S4).

A similar situation is observed for some isolates with 1233 nt CP gene, which cluster differently due to the alternative indel pattern IND5ins-IND6del they possess (Figure 2, Figure S1; Table 1) compared to the IND1ins (Figure S4). It is noteworthy that the phylogenetic analysis based on aa sequences was in full agreement with all the above results (Figure S9).

Based on the above data, it is also clear that the number of different CP sizes does not determine an equal number of phylogenetic subclusters but is correlated to specific characteristics of indels. For example, 1233 nt CP size variants are found in three different phylogenetic groups, while the 1185 nt CP size ones show up in four diverse phylogenetic groups (Table 1, Figure S4).

### 2.10. Analysis of Selection Pressure in the ASPV CP Gene

The CP genes of plant viruses are in general under negative (purifying) selection constraints, although the strength of this purifying selection has been proposed to be modulated by the mode of transmission [31]. On the other hand, while most codons are under purifying selection, a limited number of codons under diversifying selection pressure have been identified (see, for example, [32,33]). This possibility was evaluated in the case of the CP gene of ASPV using a dataset of 112 sequences obtained by removing all recombinant isolates identified by an RDP4 analysis from the 124 selected sequences dataset since the presence of such recombinant isolates is known to increase the probability of false-positive detection. Several programs of the HyPhy package implemented on the Datamonkey server (http://www.datamonkey.org/ accessed on 10–14 December 2020) and involving either ML (FEL, SLAC, MEME) or Bayesian (FUBAR) approaches were used. No statistically significant evidence for positive selection was obtained using SLAC, MEME and FUBAR, while FEL identified codon 38 as possibly under diversifying selection (*p*-value 0.079). However, all three programs assume a constant selection pressure on individual codons along the entire phylogeny and, therefore, cannot account for the possibility that isolates belonging to different clusters may be under different selection pressures. This may explain why additional evidence for selection at the codon level was observed by Komorowska et al. [24] using a more restricted dataset.

### 2.11. Protein Co-Evolution Networks of ASPV CP

**Figure 4:**
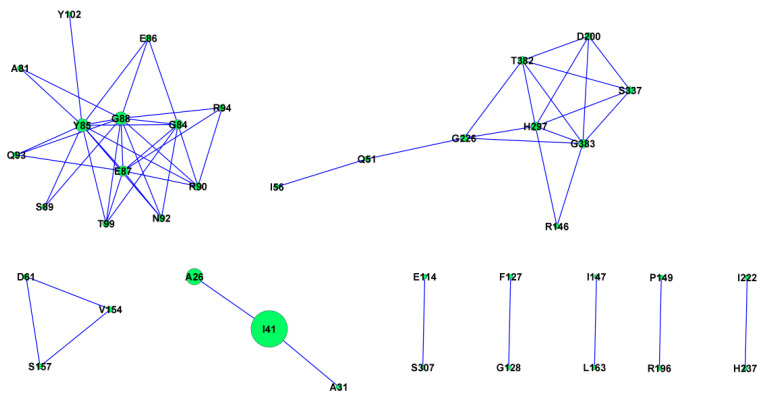
The coevolving groups and overlapping coevolving groups of amino acids were detected by CAPS2 and visualized by CYTOSCAPE. Larger nodes correspond to greater numbers of connected sites.

Protein evolution depends on intramolecular co-evolutionary networks whose complexity is correlated with functional and structural interactions among particular sites [34]. One of the key forces shaping proteins is the co-evolution of amino acid residues. Knowing which residues coevolve in a particular protein is essential to understand protein evolution, structure, and function and helps to identify substitutions that may affect its properties [35]. The presence of coevolving amino acids in ASPV CP was analyzed using the protein sequences derived from the CP gene dataset. CAPS2 analyses revealed 15 groups of coevolving amino acids (with a total of 25 aa involved). The coevolving groups, together with overlapping groups of coevolving amino acids, are presented in Figure 4. Larger nodes correspond to greater numbers of connected sites.

## 3. Discussion

The specific PCR assay using degenerate primers developed here, amplifying the full-length CP gene, allowing the in-depth study of the prevalence of ASPV and its variability in various hosts. The detection range of this method was evaluated using 180 ASPV apple and pear isolates previously detected using an assay targeting the RdRp-encoding region [13]. Primer pairs targeting the complete CP gene have been reported previously, but their detection range was not extensively validated, or they detected only a limited number of ASPV isolates [22,23,24,25,36]. Here, a mixture of two degenerate primers successfully amplified 121 ASPV isolates. Several reasons could explain that no amplification was obtained for another 59 isolates. A low viral titer and insufficient amplification efficiency may have contributed, together with the possible degradation of some samples. The alternative hypothesis is that due to genome variability, mismatches between the primers and targeted viral sequences [37] may have prevented amplification, as previously shown for other ASPV primers [38].

The only reports on ASPV detection in quince are restricted to the elucidation of the etiology of the quince deformation disease in Greece and another severe quince disease caused by an ASPV variant [6,10]. The results reported here represent the first survey conducted to study the distribution and epidemiology of ASPV in quince in Greece. Similar to the reported high infection rates in apple, pear and Rosaceae species [13,14], ASPV was prevalent in symptomatic quince (52%) with a wide geographic distribution. There was also a very strong correlation between ASPV presence and symptoms of quince deformation disease, with only one asymptomatic tree found positive to ASPV, underlining the crucial role of noncertified propagative material in ASPV spread.

To date, 25 different ASPV CP gene length variants have been reported [24]. The Greek isolates analyzed here had 5 different CP sizes, whereas the new Brazilian apple isolates and botanical pear species isolates had CP genes of, respectively, six and 11 different sizes. The 1200 nt CP size found in three botanical pear isolates (*P. pyrifolia* ct1284, *P. sinkiangensis* ct1501, *P. ussuriensis* ct481) represents a novel CP length not previously reported. The majority of ASPV isolates have a CP gene of 1191 nt (163 isolates) followed by genes of 1245 nt (78 isolates), 1233 nt (47 isolates), 1185 nt (36 isolates), 1131 (21 isolates), 1122 nt (17 isolates), 1125 nt (16 isolates), 1107 nt (11 isolates), 1104 (10 isolates), 1140 nt (5 isolates), whereas other CP sizes are recorded in between one and four isolates. As reported previously by Komorowska et al. [24], this variation in CP size is due to abundant indel polymorphisms affecting the 5′ half of the ASPV CP gene. The isolates analyzed here collectively showed a total of two insertions and seven different deletions than a reference 1191 nt long CP gene. The present analysis, with the inclusion of new hosts, suggests that there is no or limited correlation between the presence of specific indels with (i) host preference [24] and (ii) the country of origin.

Sequence analysis revealed large diversity (up to 37% nt divergence), mainly observed at the N-terminus of the CP, following previous studies [20,21,22,23,24,39]. It has been previously observed that the N-terminus of ASPV CP variants belonging to different subgroups greatly varied in length and sequence, which may suggest that the CP N-terminus is involved in interactions with host factors [23,40]. Recently, it was also shown that ASPV CP functions as a symptom determinant and differences in aa in the CP N-terminus might affect the ability of variant CP subunits to aggregate in *Nicotiana benthamiana* [40]. The addition of other hosts (botanical pear species, quince) enriched the analysis of the average divergence values of intra- and inter-groups. This showed that contrary to a previous report [24], the highest contribution to the observed variability is due to differences between isolates from different hosts and, second, to differences between isolates from the same host, providing a new understanding of the forces shaping ASPV populations’ structure. Compared to the study of Komorowska et al. [24], in which only Polish isolates were analyzed, using a larger dataset with sequences from additional hosts and from different countries also suggests the country of origin as another parameter structuring the observed ASPV variability. Chinese isolates shared the highest intra- and inter-group divergence values, but this may also reflect a potential confounding effect since the most divergent host group is that of botanical isolates, which are also of Chinese origin. It is, therefore, unclear whether the higher diversity of Chinese isolates reflects their geographical origin, their host origin or a combination of these two factors.

Previous studies performed largely at the country level or in single hosts have only reported results regarding the phylogenetic grouping of ASPV isolates based on the host of origin, and those were, at times, conflicting [20,21,23,24,39]. The present detailed work in which all (or representatives from all) the available isolates have been included revealed that the clustering of ASPV isolates was influenced at a first level by the CP size of isolates. However, a more detailed analysis revealed that the phylogeny (nt and aa) reflected indel polymorphisms rather than CP size per se. This correlation was related to the type, position, variability and pattern of indels, which often represent hallmarks of specific phylogenetic subclusters. This is confirmed by the more distant relationships that exist between isolates of the same CP size but having different indel characteristics. These results follow the first detailed study of indels in ASPV [24], which similarly identified an association between indels and phylogeny, except for IND2del, IND5ins/IND6del, IND9del and IND3del for which either no or very few Polish isolates were available, preventing a clear conclusion. The tight phylogeny observed between isolates of different CP sizes sharing the same particular indels further supports the correlation shown in Figure S4.

Our results also revealed an incomplete correlation between host of origin (apple, pear/quince, botanical) and phylogeny using either nt or aa sequences. On the other hand, there was only a low or no impact of the country of origin on the phylogeny of ASPV isolates besides the absence of Greek and Polish isolates from cluster III. However, this is possibly explained by the fact that cluster III contains only HTS-derived or genome walking-derived sequences and no RT–PCR amplified sequences. This apparent geographical effect could, therefore, more likely reflect an inability of the PCR techniques used to amplify isolates belonging to that particular cluster (see above). Indeed, an alignment of the primers used here and cluster III sequences indicated mismatches in the 3΄ end of the forward primer, which probably could prevent amplification, possibly explaining at least partially why only 121 isolates could be amplified out of the 180 tested. Therefore, the non-amplified Greek isolates may represent potential isolates of this group and result from a low prevalence or absence of such isolates in Greek apples, pears and quinces.

Recombination is considered another important factor for virus evolution, and here, we first report recombination events in the ASPV CP gene for new hosts (quince, botanicals) (Table 2). The presence of the same indels between isolates of different CP sizes could be due to recombination. For instance, Komorowska et al. [24] reported in their study that a KY176820 isolate was produced through recombination by an isolate, which contained a deletion (KY213867) and an unknown isolate and here we identified the KY176820 as potential recombinant of KY213867 and D21829. An interesting observation, with the large set of isolates used here, is the presence of numerous repetitions of a protein motif SxP (and various higher-order combinations of it, such as SxPxSxP, SxPxxSxP) in the first 64 aa of the ASPV CP N-terminal region. These repeated motifs derive from imperfect repeated nucleotide TCnnnnCC, which is present from 2 (for example, FR694929) to 6 (for example, D21829) copies, and it is tempting to speculate that these short incompletely conserved repetitions may contribute, through virus replication or imperfect recombination, to the intense indel polymorphism marking the ASPV CP N-terminal region.

Our study also revealed that some CP amino acids do not evolve in isolation but form co-evolutionary networks, which may engage functional sites and their neighbors in the protein structure. Some of the identified coevolving sites are spatially close, supporting a functional and maybe structural complexity.

For ASPV, the only known dissemination mode is through the infected propagative material, possibly raising the existence of new variants through recombination. It is noteworthy that for pome fruits, the propagation practices vary between the different hosts so that apples are grafted on apple rootstocks which are different from the rootstocks used for pear or quince. This may contribute to the partial structuring of ASPV populations by the isolation host. By contrast, quince has been a common rootstock for pear cultivars, which may explain the closer relationships between quince and pear isolates. However, the phylogenetic and recombination analyses suggest the existence of exchanges of isolates between apple and pear/quince, for which a potential mechanism remains to be proposed. The same need to explain exchanges of isolates between seemingly separated hosts exists in the isolates identified in botanical pear species. While many of these isolates cluster in divergent cluster III with only a few isolates coming from cultivated hosts, other isolates from botanical pears clearly point to exchanges and recombination between host groups, for which there is no agricultural correlation. The existence of a yet unidentified ASPV transmission mechanism, therefore, remains an open question, and so is the possibility that these symptomless wild or ornamental hosts may occasionally serve, as shown for other viruses [41], as reservoirs for ASPV.

## 4. Materials and Methods

### 4.1. Viral Isolates and Development of a Two-Step RT–PCR Assay for ASPV Detection

During a survey conducted in 19 geographical districts in Greece, 245 samples were collected (134 apples and 111 pears) [13]. The samples found positive using an ASPV-specific nested RT–PCR assay amplifying part of the RdRp gene [6] (123 apple and 57 pear samples) were used in the present study. Moreover, 48 quince samples were collected from 10 different districts of Greece (Table S1). Twenty-five were collected because they showed typical quince fruit deformation disease symptoms associated with ASPV [6], while 23 others were randomly collected from the same or different areas and did not show specific symptoms.

Total RNAs were isolated from a mixture of tissues (bark, leaves and petioles) as previously described [6] and further used as a template in a two-step RT–PCR assay in which an oligo d(T)_18_ primer was used to prime cDNA synthesis according to manufacturer’s instructions. The ASPV-specific primers used were designed from multiple alignments of ASPV genomic sequences. To overcome possible genome variability between isolates, degenerate primers were designed. The SPCPup sense primer (5΄-CAATCATGACTTCCAATGGATCYCA-3΄) was designed to start in a conserved region of five nucleotides (nt) before the CP gene start codon. Two antisense primers were designed to bind in the 3΄UTR region, the SPCPdw1 (5΄-TGCTTTAGCTTATTTTTGTTTTAACTAGA-3΄) leading to the inclusion of a ~125 nt fragment of the 3΄UTR in the amplicon and the SPCPdw2 (5΄-CCTAAYYGGGRCGGCTAWGTG-3΄) leading to the inclusion of a ~85 nt fragment, respectively. Moreover, an oligo d(T)_28_ primer was also used as an additional downstream primer to hybridize with the 3΄ poly(A) tail of ASPV. Briefly, the 25 μL PCR reaction mix contained Green-Go Taq Flexi buffer (Promega), 1.5 mM MgCl_2_, 5% DMSO, 0.2 mM dNTPs, 0.4 μΜ SPCPup primer, 0.2 μM SPCPdw1 or 1 μM SPCPdw2 or 1 μM oligo d(T)_28_ (in different PCR reactions), 1 U Go-Taq polymerase (Promega, Madison, WI, USA) and 2μL of cDNA. After PCR optimization, the following PCR cycling scheme was retained for all primer combinations: 94 °C for 4 min, 40 cycles of 95 °C for 30 s, 55 °C for 30 s, 72 °C for 100 s and a final step of 72 °C for 5 min. The size of the amplified PCR products is expected to vary from 1215 to 1410 bp, depending on the primer pair used. Moreover, a single PCR was also performed simultaneously using both antisense primers under the same above conditions.

### 4.2. Restriction Fragment Length Polymorphism (RFLP) Analysis

Amplicons obtained from all the apple, pear and quince samples were subjected to RFLP analysis as a preliminary screen to identify the most diverse isolates. Based on previously available ASPV CP sequences, three restriction enzymes were chosen, *MspI*, *RSaI* and *Sau3AI* (New England BioLabs, Ipswich, MA, USA), based on their cleavage site frequency in other isolates. Five units of each restriction enzyme (with its corresponding reaction buffer) were used with 8.5 μL of PCR products in a final volume of 10 μL. After an incubation time of 12 h, the reaction products were analyzed in a 2% agarose gel.

### 4.3. Cloning and Sequence Analysis

A total of 14 isolates (five from apple, five from quince and four from pear), which showed distinct RFLP profiles, were chosen for further analysis. The PCR amplicons were purified using a matrix gel extraction system (Marligen Bioscience, Ijamsville, MD, USA) and cloned into the TOPO-II vector (Invitrogen, Thermo Fisher Scientific, Waltham, MA, USA). Positive clones were Sanger sequenced in both orientations using the universal M13 forward and reverse primers.

### 4.4. Identification of Additional ASPV CP Gene Sequences from High-Throughput Sequence Data

Additional ASPV CP gene sequences were obtained from two sources. The first is Illumina HiSeq X RNAseq data obtained from three Brazilian apple trees that showed multiple infections by ASPV isolates. While the presence of ASPV and of *Buyna*-like viruses in these trees has been previously described [42], a more detailed analysis of the contigs from each tree allowed in each case the recovery of several complete CP gene sequences.

In parallel, publicly available transcriptomic RNAseq data for botanical *Malus* and *Pyrus* available as sequence reads archive (SRA) in GenBank were screened by Blast analysis. Those SRAs with significant ASPV presence (only observed in *Pyrus* SRAs) downloaded for further analysis. Upon de novo assembly using CLC Genomic Workbench 20.0, BLASTx was used to identify the contigs containing a full-length ASPV CP gene, which were retained for sequence and phylogenetic analyses.

### 4.5. Molecular Variability and Recombination Analysis

To estimate the divergence levels, multiple alignments of nucleotide nt or translated aa sequences were performed using the ClustalX [43] and/or Muscle [44] software implemented in MEGA X [43]. The multiple sequences (nt) were aligned by codons for the phylogenetic analysis after removing the gap positions using the Muscle software as implemented in MEGA X [44,45]. A preliminary evaluation of the 429 available ASPV CP sequences in the NCBI (from apple, pear, hawthorn and from 10 countries: China, Poland, Germany, Brazil, India, Japan, South Korea, UK, USA, Czech Republic) was conducted in which the isolates with a non-functional CP gene were removed. In a second step, isolates with more than 90% nucleotide identity were excluded along with the isolates of the same origin and host, which were placed in the same phylogenetic clusters so that in each case, a single representative isolate was retained. The same was applied to the new sequences from Brazilian apples and from botanical pear species SRAs. This yielded a final dataset composed of 124 sequences corresponding to the 14 Greek isolates, 69 ASPV sequences (of different CP-length, host and origin) from GenBank, 24 sequences from wild and ornamental *Pyrus* species SRAs and 17 sequences from Brazilian apple samples. In addition, this nucleotide sequence dataset was used to calculate average values of intra- and inter-groups divergence using host-based (four types: apple, pear, quince, botanical species) or country of origin-based groups (five origins: Brazil, China, Greece, Poland and Rest of the world) using MEGA X [45]. Before phylogenetic analyses, the occurrence of potential recombination events was evaluated using various methods (RDP, GENECONV, Chimaera, MaxChi, BootScan, SiScan and 3Seq) implemented in RDP4 [46]. Recombination events were considered significant if detected by five or more methods with a probability *p*<0.05 in addition to phylogenetic evidence of recombination. Moreover, a split decomposition analysis of the above dataset was performed with SplitsTree4 [47] to obtain a split network representing ambiguous signals and further confirmation of the potential recombination events.

### 4.6. Phylogenetic Analysis and Evolutionary Networks

Sequence alignments were used for inferring phylogenetic relationships and the construction of NJ and ML trees using MEGA X. The best nucleotide substitution model was calculated using the ModelTest program implemented in MEGA X. Specifically, the general time-reversible (GTR) model [48] with a gamma distribution (parameter α = 0.8273) of variable and invariant sites (*G+I*) (*I* = 0.1436) was used to compute the ML tree of the 124 ASPV CP gene nt sequences, whereas the JTT and amino acid frequencies (*F*) model [49] with *G+I* (parameter α = 0.4851; *I* = 0.1438) was used to compute the ML tree of the 124 ASPV CP aa sequences. Confidence in the branch points was assessed by the bootstrap method with 1000 pseudorandom replicates [50]. Evolutionary networks were studied by parsimony phylogenetic networks, constructed with SplitsTree4 [47] using the same alignment of the 124 ASPV nt sequences used in the recombination and phylogenetic analyses. The significance of nodes was evaluated by 1000 pseudo-replicates.

### 4.7. Inference of Selection Pressure in the ASPV CP Gene and Co-Evolution of Amino Acids

To try to identify codons under positive selection (i.e., with a dN/dS ratio significantly higher than 1) in the ASPV CP gene, ML or Bayesian approaches implemented in the HyPhy package on the Datamonkey server (http://www.datamonkey.org/, accessed on 3 March 2021) were used. As the presence of recombinant isolates leads to an increase in false positives detection, all recombinant isolates identified using RDP4 [46] were removed from the dataset before selection analysis. Multiple alignments of the remaining sequences were then obtained using Muscle software as implemented in MEGA 7. In HyPhy, the single-likelihood ancestor counting (SLAC) [51], fixed effects likelihood (FEL) [51], mixed-effects model of evolution (MEME) [52] and fast unconstrained Bayesian approximation (FUBAR) [53] methods were used.

The presence of coevolving sites in the proteins translated from the analyzed dataset was investigated using CAPS2 (https://caps.tcd/ie, accessed on 3 March 2021) [54], and the relationships between identified covarying sites were visualized using CYTOSCAPE version 3.8.1 [55].

## Figures and Tables

**Table 1:**
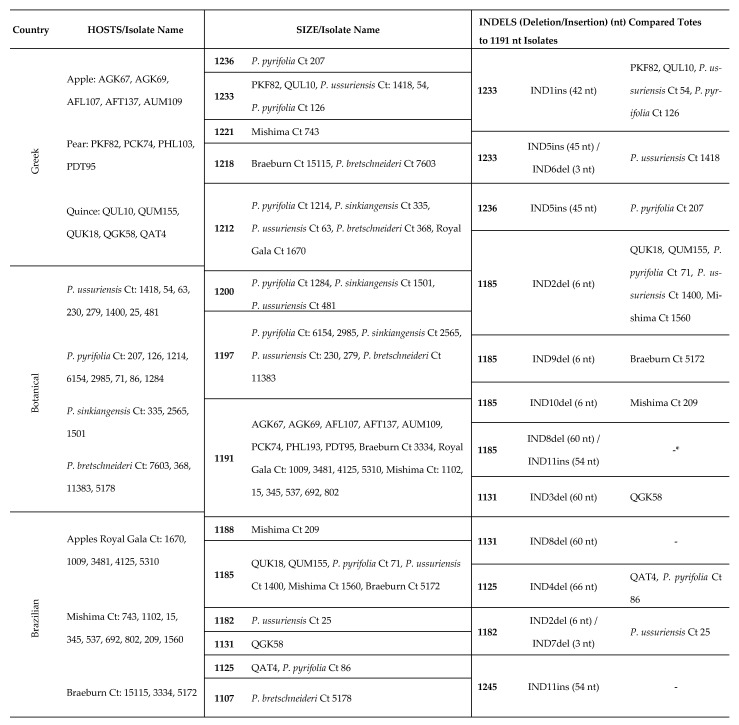
List of isolates from Greece, Brazil and botanical species studied, according to their host type, size and presence of indels cited. * none of the isolates analyzed in the present study possess these indels.

**Table 2:** List of potential recombination events involving ASPV CP gene sequences detected by RDP, GENECONV, Chimaera, MaxChi, BootScan, SiScan and 3Seq methods using RDP4 program.

Country	Host	Recombinant Isolate	Major Parent (Host, Country)	Minor Parent (Host, Country)	No. of Methods with *P* < 0.05
Greek	Quince	QUL10	KY243369 (pear, Poland)	KY176823 (apple, Poland)	6
	Pear	PCK74	KY429181 (pear, Poland)	KY176815 (pear, Poland)	7
	Pear	PHL193	KY429181 (pear, Poland)	KY176815 (pear, Poland)	7
Botanical -China	*P. ussuriensis*	Ct54 (SRX670013)	JX673812 (pear, China)	JX673811 (pear, China)	7
Brazilian	Apple cv Mishima	Ct15	KR815875 (pear, India)	AF345892 (pear, Poland)	7
	Apple cv Royal Gala	Ct1670	HE963831 AGCaV (apple, Canada)	Braeburn Ct3334 (apple, Brazil)	6
Other	Apple	KM73720	KY429181 (pear, Poland)	AF345892 (pear, Poland)	6
	Apple	KR815876	KF319056 (apple, Germany)	MK923754 (apple, Brazil)	7
	Apple	KF319056	KR815875 (pear, India)	AF345892 (pear, Poland)	7
	Pear	KY176820	KY213867 (pear, Poland)	D21829 (pear)	6
	Apple	KY176808	JX673812 (pear, China)	JX673811 (pear, China)	7
	Pear	HM325767	JX673812 (pear, China)	JX673811 (pear, China)	7
	Pear	AF345894	AF345895 (pear, Poland)	AF491929 (Pear, Poland)	7 [20]

**Table 3:** Average nucleotide pairwise intra-group and inter-group divergence values (±standard deviation) for country of origin groups (upper table) and for a host of origin groups (lower table). ^1^ The number of isolates of each group is indicated in parenthesis, ^2^ RoW: rest of world (8 countries: India, Germany, Czech Republic, South Korea, Taiwan, Japan, Canada, UK).

Greece ^1^ (14)	Brazil (22)	Poland (31)	China (38)	RoW ^1,2^ (17)	Country
0.221 ± 0.006	0.254 ± 0.007	0.205 ± 0.007	0.275 ± 0.007	0.244 ± 0.007	Greece
-	0.256 ± 0.007	0.247 ± 0.007	0.286 ± 0.007	0.257 ± 0.007	Brazil
-	-	0.185 ± 0.006	0.267 ± 0.007	0.234 ± 0.007	Poland
Quince	0.174 ± 0.007	-	0.290 ± 0.008	0.290 ± 0.008	China
Botanicals	0.271 ± 0.008	0.298 ± 0.008	-	0.260 ± 0.007	RoW ^1^
Pear	0.197 ± 0.006	0.274 ± 0.007	0.207 ± 0.006	-	
Apple	0.244 ± 0.007	0.287 ± 0.007	0.245 ± 0.007	0.248 ± 0.008	
**Host**	**Quince ^1^ (5)**	**Botanicals (28)**	**Pear (35)**	**Apple (54)**	

## Data Availability

The data presented in this study are available in this article.

## Supplementary Materials

The following are available online at https://www.mdpi.com/article/10.3390/plants10050917/s1, Figure S1: Nucleotide (nt) sequence alignment of the 14 Greek isolates and some botanical isolates studied here with other available ASPV CP sequences. The alignment shows the first 176 nt of the N-terminal. The alignment was conducted using the CLUSTAL X program; Figure S2. Decomposition phylogenetic networks constructed using an alignment of 124 full-length ASPV CP nucleotide sequences. The network was constructed using SplitsTree 4.0; Figure S3: NJ phylogenetic tree conducted in MEGA X using full-length ASPV CP nucleotide sequences. The analysis involved the 14 ASPV Greek isolates, reported with their isolate name, host type and country of origin. Branches with <80% bootstraps were collapsed. The scale bars represent a genetic distance of 0.05; Figure S4. ML phylogenetic tree (classical shape) conducted in MEGA X using full-length ASPV CP nucleotide sequences. The analysis involved 124 ASPV isolates, with a total of 1316 positions in the final dataset. All GenBank isolates are reported with their accession numbers, isolate name, host type and country of origin. The isolates from this study are indicated by their isolate name, host and country of origin. In parenthesis is given the size of the CP gene. Isolates, which possess indels, are indicated last by indels code name. Indels are also color-coded: green: IND1ins, pink: IND2del, orange: IND3del, blue: IND4del, brick red: IND5ins, light blue: IND8del, purple: IND9del, gray: IND10del. By red and brown color are indicated, respectively isolates of different CP-size with deletions of 69 and 87 nt. I-III and a-h represent, respectively, the clusters and subclusters. Branches with <70% bootstraps were collapsed. The scale bars represent a genetic distance of 0.05; Figure S5: The ML phylogenetic tree (radical shape) of the CP nucleotide sequences, conducted in MEGA X. The identified clusters (I to III) and subclusters (a to h) are easily visible in this tree shape. Greek isolates are reported by their isolate name and a black dot. The scale bars represent a genetic distance of 0.2; Figure S6: The ML phylogenetic tree (radical shape) of the CP nucleotide sequences, conducted in MEGA X. The isolates are indicated by their accession number or isolate name, host and country of origin. The sizes of the CP gene are given in a parenthesis and also are color-coded: green: 1233, pink: 1185, orange: 1131, blue: 1125, dark green: 1221, light blue: 1245, purple: 1218, gray: 1194, fuchsia: 1212, red: 1191, yellow: 1200 and brown: 1197. Branches with <70% bootstraps were collapsed. The scale bars represent a genetic distance of 0.05. A classical shape tree can be examined in Figure S4; Figure S7: The ML phylogenetic tree (radical shape) of the CP nucleotide sequences, conducted in MEGA X. The isolates are indicated by their accession number or isolate name, host and country of origin. The isolates are color-coded based on the country of origin: pink: Poland, blue: Greece, green: China, red: Brazil, and orange: rest of world. Branches with <70% bootstraps were collapsed. The scale bars represent a genetic distance of 0.05. A classical shape tree can be examined in Figure S4; Figure S8: The ML phylogenetic tree (radical shape) of the CP nucleotide sequences, conducted in MEGA X. The isolates are indicated by their accession number or isolate name, host and country of origin. The isolates are color-coded based on the method of detection: green: HTS, red: PCR and specifically with square PCR amplified isolates by one primer pair, whereas with circle the full-length isolates by PCR primer walking. Branches with <70% bootstraps were collapsed. The scale bars represent a genetic distance of 0.05. A classical shape tree can be examined in Figure S4; Figure S9. ML phylogenetic tree conducted in MEGA X using full-length ASPV CP amino acid sequences. The analysis involved 124 ASPV isolates, with a total of 438 positions in the final dataset. All GenBank isolates are reported with their accession numbers, isolate name, host type and country of origin. The isolates from this study are indicated by their isolate name, host and country of origin. In parenthesis is given the size of the CP gene. Isolates, which possess indels, are indicated last by indels code name. Indels are also color-coded: green: IND1ins, pink: IND2del, orange: IND3del, blue: IND4del, brick red: IND5ins, light blue: IND8del, purple: IND9del, gray: IND10del. By red and brown color are indicated, respectively isolates of different CP-size with deletions of 69 and 87 nt. I-III and a-g represent, respectively, the clusters and subclusters. Branches with <75% bootstraps were collapsed. The scale bars represent a genetic distance of 0.10; Figure S10. Parsimony phylogenetic networks constructed using an alignment of 124 full-length ASPV CP nucleotide sequences. The network was constructed using SplitsTree 4.0.
