# Supplementary material for: Molecular Characterization of the Coat Protein Gene of Greek Apple Stem Pitting Virus Isolates: Evolution through Deletions, Insertions, and Recombination Events
Source: Plants (Basel). 2021 May 3;10(5):917. doi: 10.3390/plants10050917 (PMC8147640; doi:10.3390/plants10050917)
Supplement: Supplementary file 1 [file plants-10-00917-s001.zip › plants-1192196-supplementary/Supplementary Material/Table S1.pdf]

Table S1. List of Greek districts of the collected quince samples and incidence of apple stem pitting virus.

Geographic area	Cultivar	No of samples collected	No of samples with symptoms	ASPV infected
Chalkidiki	Unknown	5	4	4
	Milokidono	3	0	0
Fthiotida	Gigas	4	2	2
Imathia	Unknown	4	2	2
Ioannina	Unknown	4	2	2
Karditsa	Afrata	4	0	0
Kozani	Gigas	5	4	3
Lakonia	Gigas	4	0	0
Larissa	Unknown	5	2	3
Magnisia	Unknown	5	5	5
Thesaloniki	Afrata	5	4	4
Total		48	25	25
