# Supplementary figures and images for: Molecular Characterization of the Coat Protein Gene of Greek Apple Stem Pitting Virus Isolates: Evolution through Deletions, Insertions, and Recombination Events
Source: Plants (Basel). 2021 May 3;10(5):917. doi: 10.3390/plants10050917 (PMC8147640; doi:10.3390/plants10050917)

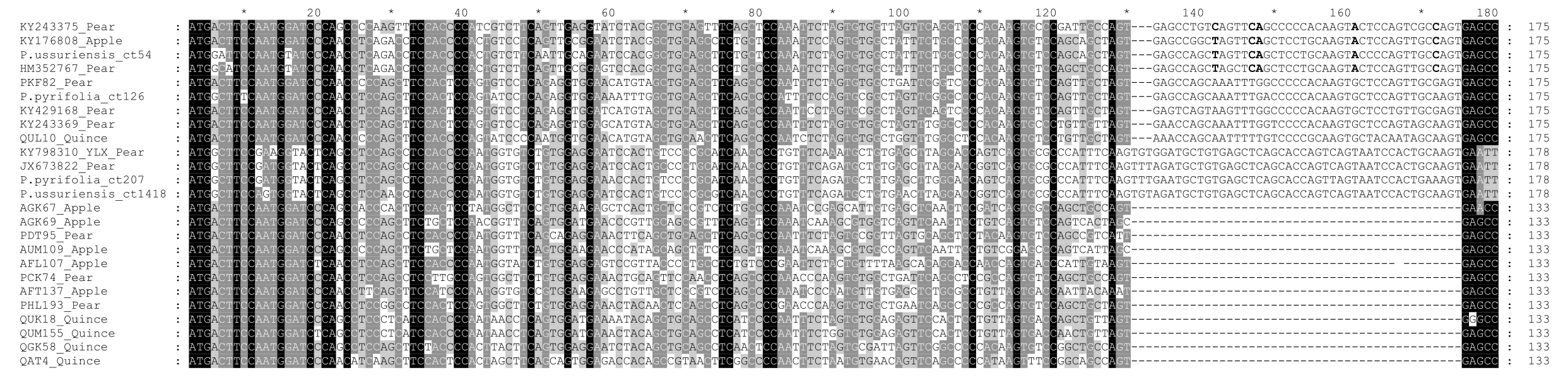

Supplement: Supplementary file 1 [file plants-10-00917-s001.zip › plants-1192196-supplementary/Supplementary Material/Figure S1.png]

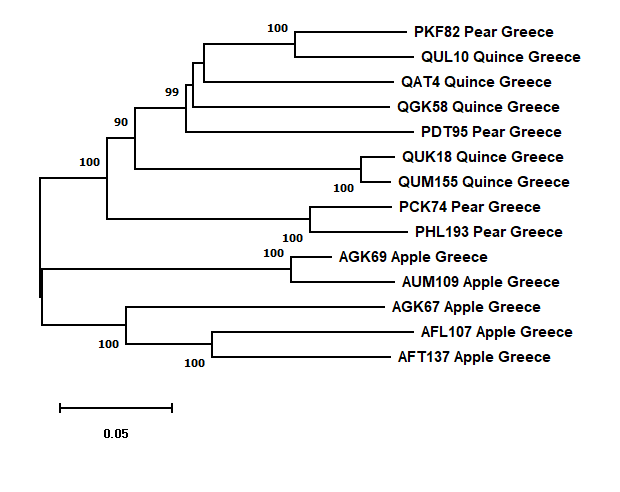

Supplement: Supplementary file 1 [file plants-10-00917-s001.zip › plants-1192196-supplementary/Supplementary Material/Figure S3.png]

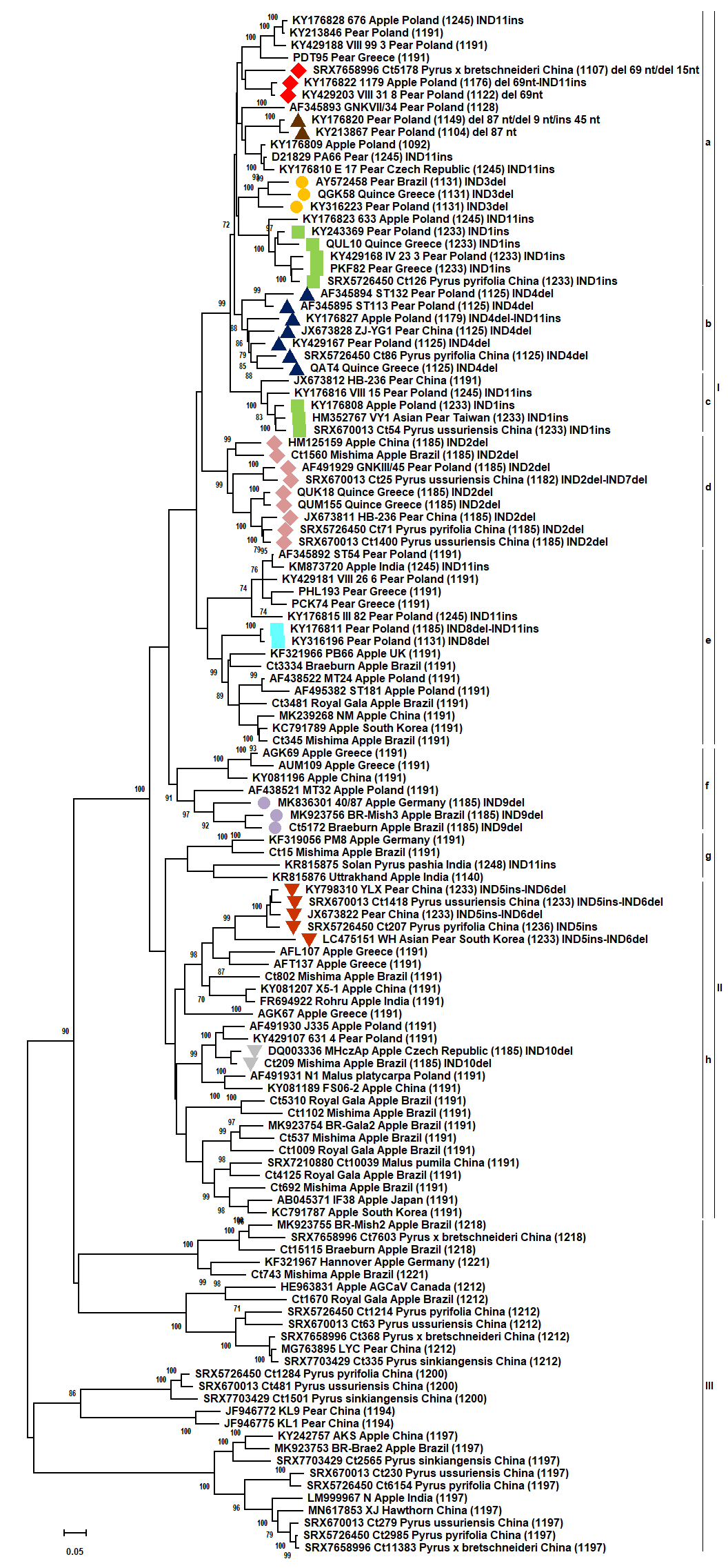

Supplement: Supplementary file 1 [file plants-10-00917-s001.zip › plants-1192196-supplementary/Supplementary Material/Figure S4.png]

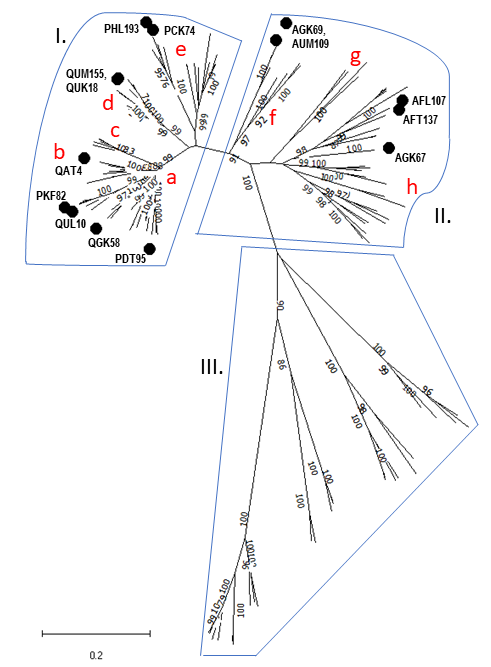

Supplement: Supplementary file 1 [file plants-10-00917-s001.zip › plants-1192196-supplementary/Supplementary Material/Figure S5.tiff]

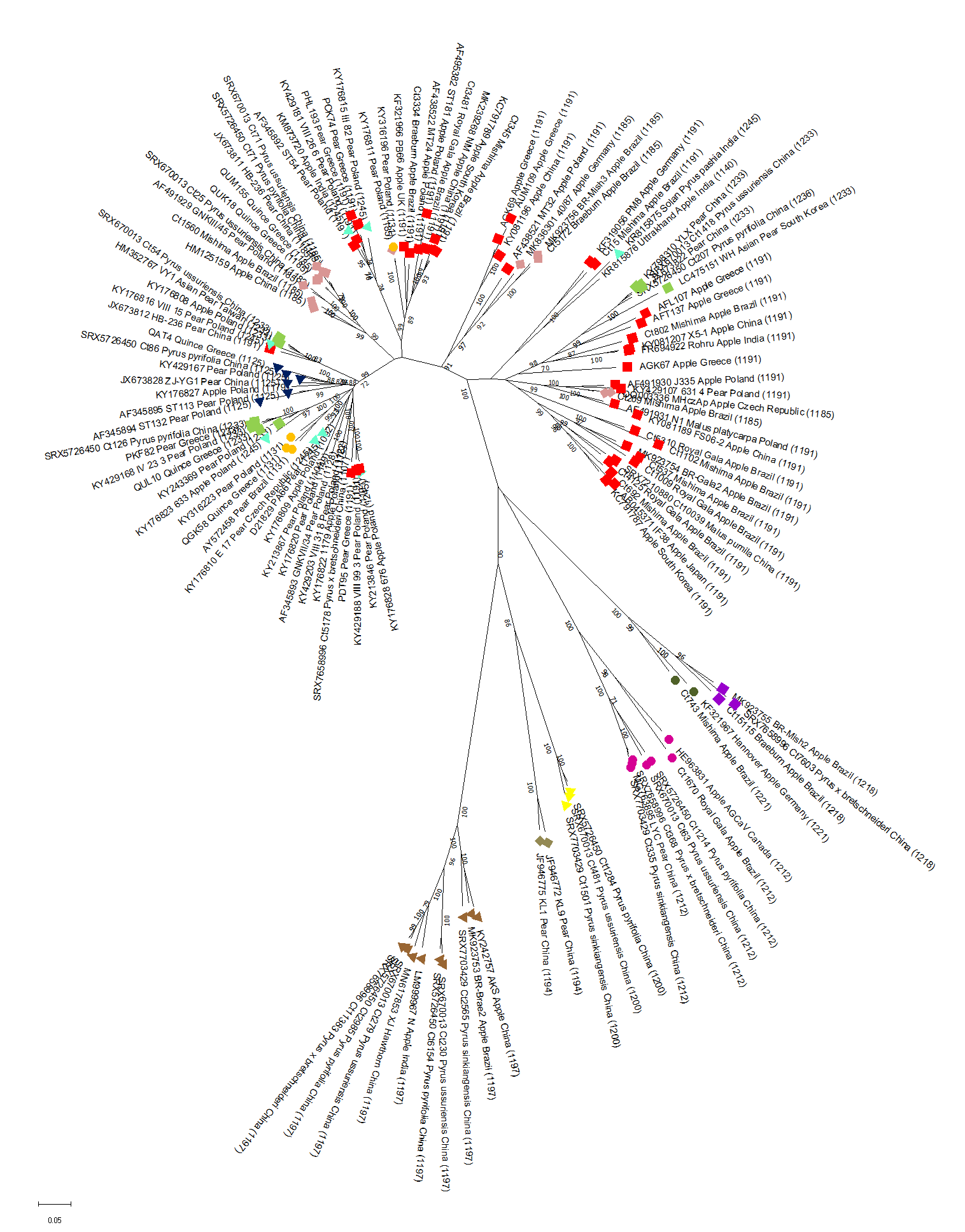

Supplement: Supplementary file 1 [file plants-10-00917-s001.zip › plants-1192196-supplementary/Supplementary Material/Figure S6.png]

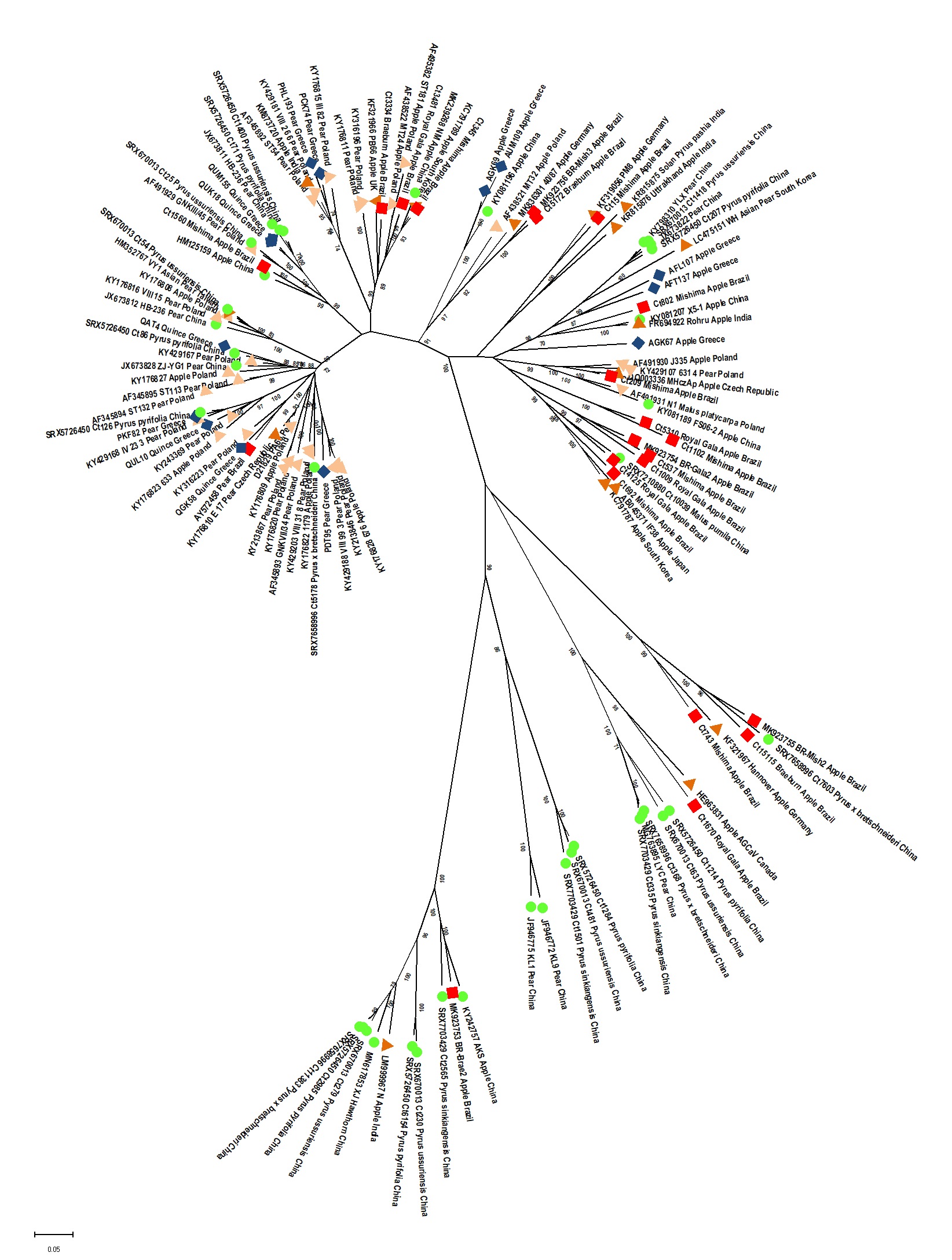

Supplement: Supplementary file 1 [file plants-10-00917-s001.zip › plants-1192196-supplementary/Supplementary Material/Figure S7.png]

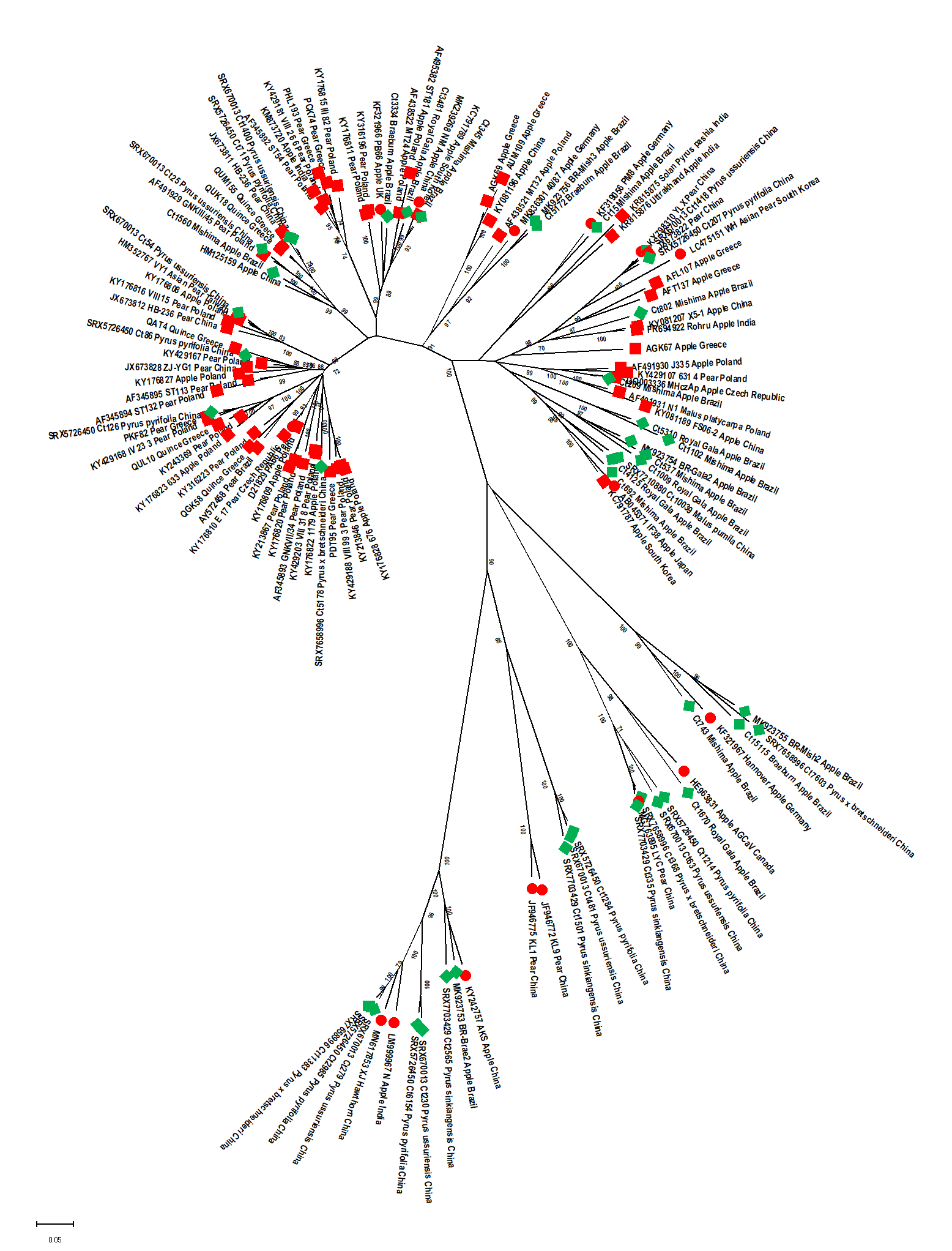

Supplement: Supplementary file 1 [file plants-10-00917-s001.zip › plants-1192196-supplementary/Supplementary Material/Figure S8.png]

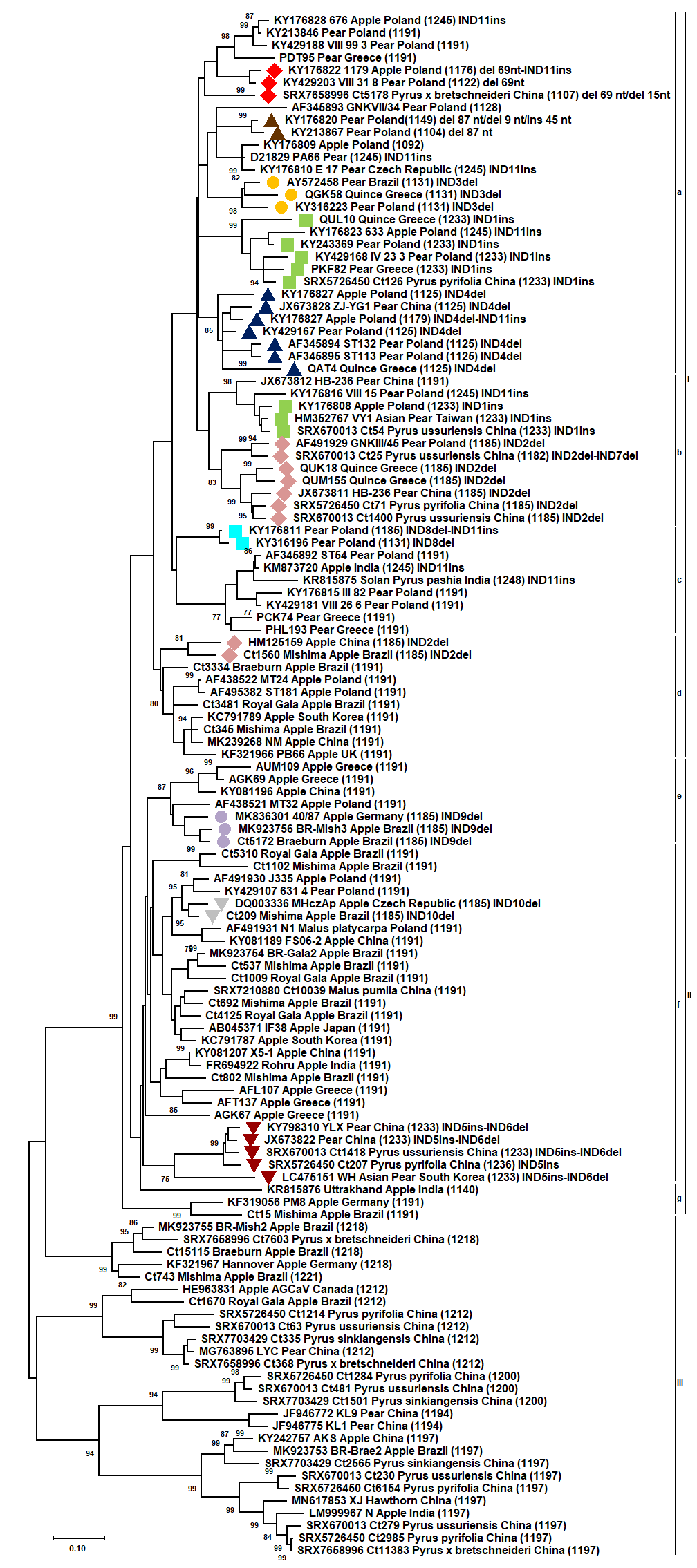

Supplement: Supplementary file 1 [file plants-10-00917-s001.zip › plants-1192196-supplementary/Supplementary Material/Figure S9.png]
